# Predictors of etiology and drug resistance in children with new‐onset focal seizures

**DOI:** 10.1002/epi4.70179

**Published:** 2026-01-16

**Authors:** Byoung Chan Lee, Russell C. Dale, Elizabeth H. Barnes, Shekeeb S. Mohammad, Sachin Gupta, Chong Wong, Deepak Gill, Kavitha Kothur

**Affiliations:** ^1^ The Children's Hospital at Westmead Clinical School The University of Sydney Sydney New South Wales Australia; ^2^ TY Nelson Department of Neurology and Neurosurgery The Children's Hospital at Westmead Sydney New South Wales Australia; ^3^ Kids Neuroscience Centre, the Children's Hospital at Westmead Sydney New South Wales Australia; ^4^ NHMRC Clinical Trials Centre, Faculty of Medicine and Health The University of Sydney Sydney New South Wales Australia; ^5^ Department of Neurology Westmead Hospital Sydney New South Wales Australia

**Keywords:** drug resistance, etiology, explosive onset, focal seizure, new onset

## Abstract

**Objective:**

To examine the clinical features of new‐onset focal seizures in children and investigate clinical associations and predictors of underlying etiology and drug resistance.

**Methods:**

Data were gathered from The Children's Hospital at Westmead admissions for patients aged 1 month to 18 years who presented with new‐onset focal seizures between 2018 and 2022 (*n* = 140). Seizure characteristics, etiology, clinical comorbidities, investigations, and antiseizure medications were analyzed. Clinical associations between etiologies and comorbidities/treatment outcomes were investigated using nonparametric tests and hierarchical cluster analysis. Multivariable logistic regression was performed to identify predictors of drug resistance.

**Results:**

The median age of seizure onset was 4.7 years (IQR 1.9–8.1). The etiologies included unknown (*n* = 53, 39%) followed by structural (*n* = 36, 26%), self‐limited childhood focal epilepsy (*n* = 21, 15%), genetic (*n* = 12, 9%), inflammatory (*n* = 12, 9%), and metabolic (*n* = 3, 2%). The explosive seizure‐onset seizures (*p* = 0.04), focal neurological abnormalities (*p* = 0.04), younger age at seizure onset (*p* = 0.01), abnormal neuroimaging findings (*p* < 0.001), and drug resistance (*p* < 0.001) were associated with known etiology. Regression analysis showed the drug resistance risk increased with the presence of known genetic (OR 6.7; 95% CI 1.6–31.8), structural (OR 6.4; 95% CI 2.3–19.5), and inflammatory (OR 4.6; 95% CI 1.0–21.2) etiologies.

**Significance:**

Our study examines the important associations and predictors of etiology and drug resistance in children with new‐onset focal seizures. The significance of known etiologies as risk factors for drug resistance promotes the need for improved monitoring and etiology‐driven treatment.

**Plain Language Summary:**

This study looked at children who had focal seizures for the first time. In many cases, the cause was unknown, but a large portion was linked to structural brain changes, childhood epilepsies that usually resolve, or genetic, inflammatory, and metabolic conditions. Children with a known cause, especially genetic, structural, or inflammatory, were more likely to have seizures that did not improve with anti‐seizure medications. Identifying the cause early can help doctors choose better treatments and provide closer monitoring for patients.

**Key points**

- Explosive seizure onset, focal neurological deficits, younger age, and abnormal neuroimaging were associated with known epilepsy etiologies.
- We highlight genetic, structural, and inflammatory etiologies as drug resistance predictors, enabling close surveillance of this subgroup.
- The prevalence of unknown etiology supports further investigation in this group.

## 1 INTRODUCTION

New‐onset seizures are one of the most common neurological problems in children, where there is an estimated lifetime risk of 8% with approximately 4% developing epilepsy in their lifetime.^1^ The predominant seizure onset in children is focal seizures, which are seen in 61%–68% of new epilepsy in population‐based studies.^2^, ^3^ The incidence rate of focal epilepsy is 32–38 per 100 000 persons per year and is highest in earlier ages.^2^, ^3^

Focal seizures have a wide range of causes, from self‐limited epilepsy syndromes with normal imaging to more complex conditions like cortical malformations, brain injuries, vascular abnormalities, and neuroimmunological disorders, some of which may be refractory.^4^ Due to the diverse nature of focal seizures in children and adolescents, tailored evaluation and treatment are essential for selecting appropriate therapies and providing family guidance. Despite the differences in unique clinical characteristics and management strategies, there is a paucity of studies on new‐onset focal seizures compared to studies investigating all seizure types in children, as presented by the literature review in Table S1. The etiologies, EEG findings, neuroimaging, and comorbidities have been described for all new‐onset seizure types, but further studies are needed to explore their variations based on seizure type and epilepsy syndrome. This discrepancy is further pronounced in the limited number of studies on new‐onset seizures that transpire as refractory seizures, which are important to understand for appropriate patient management.^5^

A significant factor in understanding new‐onset focal seizures in children is their etiology, which was revised in 2017 by the International League Against Epilepsy (ILAE).^4^ The etiologies include genetic pathogenic variants, structural abnormalities, metabolic disorders, immune disorders, infections, and unknown. Identification of these etiologies in children can lead to optimal diagnosis and treatment including genetic counseling for *DEPDC5* pathogenic variants, ketogenic diet treatment for glucose transporter 1 deficiency, and surgery for hippocampal sclerosis in temporal lobe epilepsy.^6^, ^7^, ^8^ However, the etiologies for the majority of new‐onset focal epilepsy are unknown, especially in patients older than 12 months.^2^ Although there is limited information about immunological mechanisms, they have been increasingly identified as an important cause of new‐onset seizures that become refractory.^9^ Since the 2017 ILAE classification, recent advances in genetic and immunological diagnostic testing have helped improve understanding of previously unknown etiologies.

Furthermore, treatment outcomes for new‐onset focal seizures in children are limited and drug resistance, defined as failure of seizure control from at least two anti‐seizure medications (ASM), has various proportions in pediatric populations.^9^, ^10^, ^11^ In children with all epilepsy types, population studies have shown that seizure remission of at least 5 years ranges between 57% and 75%.^12^, ^13^, ^14^, ^15^ It is important to consider the drug‐resistant outcomes in these patients, as they have increased risks for psychiatric and medical comorbidities, such as seizure‐induced injuries and sudden unexpected death in epilepsy.^16^, ^17^, ^18^

To address the above knowledge gaps in new‐onset focal seizures in children, this study aimed to describe the clinical characteristics and predictors of underlying etiologies and drug resistance. This, in turn, can provide information to assist clinicians with etiology‐driven treatment decisions and improve patient outcomes.

## 2 METHODS

### 2.1 Study participants and demographics

**FIGURE 1:**
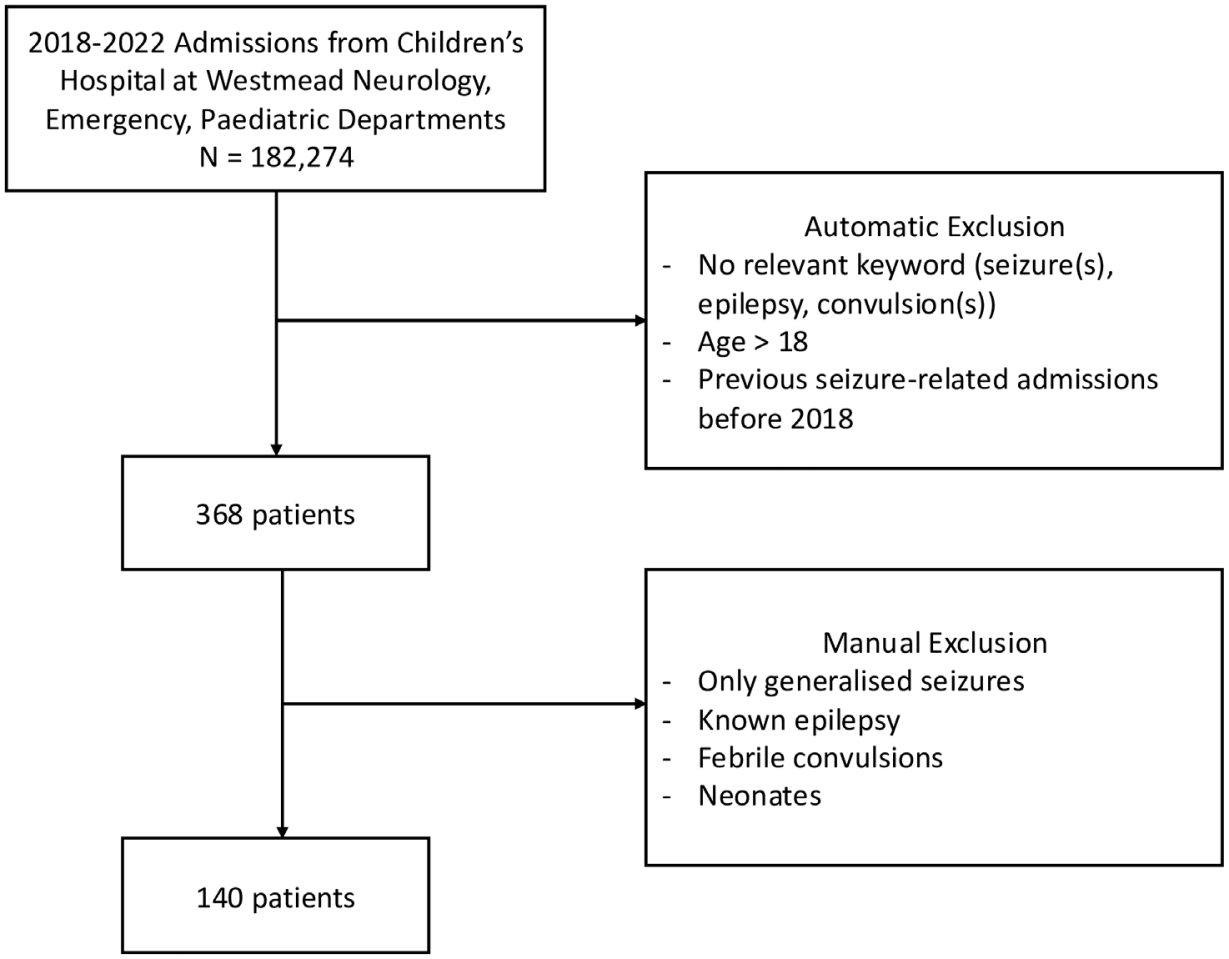
Flow chart of patient inclusion criteria.

Figure 1 shows the neurology, emergency and pediatric department admissions at The Children's Hospital at Westmead that were gathered via Management Support Analysis Unit requests (*n =* 182 274). Children (age < 18) were selected for a first seizure‐related admission between 2018 and 2022 inclusive (*n =* 368). A general keyword search (“seizure(s),” “epilepsy,” “convulsion(s)”) on the admission's presenting problem was used to determine if the admission was seizure related. One hundred forty patients with new‐onset focal seizures were subsequently identified by excluding neonates and through a manual review of the electronic medical records. Information on demographic characteristics (sex and age at seizure onset), seizure semiology, frequency, duration, etiology, family history, and neurodevelopmental comorbidities, such as learning difficulties and developmental delay, were reviewed. Investigations included EEG findings, magnetic resonance imaging (MRI), and computed tomography findings, as well as metabolic, inflammatory, and genetic tests. Medical/surgical treatment and outcomes on seizure control were also examined.

The 2017 ILAE classification was used to define seizure, epilepsy syndromes, and seizure etiology.^4^ Children were categorized by known causes such as structural, metabolic, and genetic causes. Self‐limited focal epilepsies of childhood onset (SeLFE) were defined based on the type of seizures, EEG and epilepsy clinical syndrome as per ILAE classification.^19^ The syndromes consisted of self‐limited epilepsy with centrotemporal spikes (SeLECTS), self‐limited epilepsy with autonomic seizures, childhood occipital visual epilepsy, and photosensitive occipital lobe epilepsy. Genetic etiology was defined if it had identified a monogenetic or chromosomal cause. Inflammatory etiology was used to classify immune and infectious etiologies. Explosive onset of seizures was defined as at least three seizures in the first 24 hours or at least two seizures on consecutive days. Drug‐resistant epilepsy was defined as the failure of two appropriate ASMs.^10^

This study received ethical approval from the Sydney Children's Hospital Network (ethics approval no 2023/ETH01315).

### 2.2 Statistical analysis

Descriptive statistics were used to summarize each clinical characteristic, including frequencies for categorical variables and medians and interquartile range (IQR) for continuous variables. Fisher's exact test, Chi‐square test, and the Kruskal‐Wallis test were used, where appropriate, to investigate clinical associations for etiology. Multivariable logistic regression with backward selection was performed to explore risk factors for developing drug resistance. Odds ratios (ORs) and 95% confidence intervals (CIs) were used as a measure of effect. A network graph using the Fruchterman‐Reingold force‐directed algorithm^20^ was applied to represent the degree of association between prescribed ASMs.

Hierarchical cluster analysis was used to identify clinical similarities among the patients and was weighted towards epilepsy etiology. Ward's agglomeration method^21^ with four clusters was chosen using the dendrogram and the optimal Dunn index. T‐distributed stochastic neighbor embedding was applied to visualize the high‐dimensional cluster plots in two‐dimensional space. The clinical characteristics in each cluster were visualized using a proportion‐based heatmap.

Data were analyzed using R version 4.2.1 (R Foundation for Statistical Computing, Vienna, Austria) and Python version 3.11.3 (Python Software Foundation, Delaware, United States).

## 3 RESULTS

### 3.1 Clinical characteristics

One hundred forty children with new‐onset focal seizures were identified with a median age of 4.7 years and a balanced sex distribution (Table 1). Sixty‐eight (49%) of the total cohort had neurodevelopmental comorbidities, of which language difficulty (*n =* 24, 35% of neurodevelopmental comorbidities) and autism spectrum disorder (*n* = 19, 28%) were the most common. Other neurodevelopmental disorders included attention‐deficit/hyperactivity disorder (*n* = 9, 13%), cerebral palsy (*n* = 8, 12%), hearing/vision impairment (*n* = 6, 9%), and anxiety/depression symptoms (*n* = 6, 9%). Patients often presented with multiple neurodevelopmental disorders, with 27 patients (40%) having at least two comorbidities. Family history of neurological disorders was seen in 37 patients (26% of the total cohort) of whom 28 (20%) had a family history of seizures. Forty‐five children (32%) also presented with explosive onset of seizures, of whom 10 continued to have clusters of seizures during admission. Seizure duration and circadian records revealed 43 patients (31%) experienced status epilepticus, and 44 patients (31%) had nocturnal seizures. Among associated symptoms, febrile illness was the most common (*n* = 29, 21%) followed by encephalopathy (*n* = 13, 9%). Focal neurological deficits were seen in 14 children (10%) subsequent to their seizure, with four children developing Todd's paresis. Other neurological abnormalities included prolonged hemiplegia, asymmetric clonus or tone abnormalities. Focal motor seizures were prevalent among the cohort (*n =* 114, 81%), followed by impaired awareness (*n =* 107, 76%). One hundred thirty‐seven patients (98%) also developed epilepsy.

**TABLE 1:** Demographic and clinical characteristics of children with new‐onset focal seizures.

Characteristics	Total (*n* = 140)
Sex (%)	
Male	73 (52)
Female	67 (48)
Median Age (IQR)	4.7 (1.9–8.1)
Neurodevelopmental comorbidities (%)	68 (49)
One comorbidity	41 (29)
Two comorbidities	20 (14)
Three comorbidities	7 (5)
Family history of seizures (%)	28 (20)
Explosive seizure onset (%)	45 (32)
Status epilepticus (%)	43 (31)
Nocturnal seizures (%)	44 (31)
Focal neurological abnormalities	14 (10)
Focal seizure type (%)	
Motor	114 (81)
Nonmotor	25 (18)
Impaired awareness	107 (76)
Bilateral tonic–clonic	44 (31)
Developed epilepsy (%)	137 (98)
Etiologies (%)	
Self‐limited	21 (15)
Genetic	12 (9)
Structural	36 (26)
Metabolic	3 (2)
Inflammatory	12 (9)
Unknown	53 (39)
ASM on Discharge (%)	129 (92)
Drug responsive^a^	76 (59)
Drug resistant^a^	41 (32)
Unknown^a^	12 (9)
Abnormal EEG (%)	115 (82)
Abnormal MRI/CT (%)	54 (39)

^a^ Percentage calculated from patients who received ASM on discharge.

**FIGURE 2:**
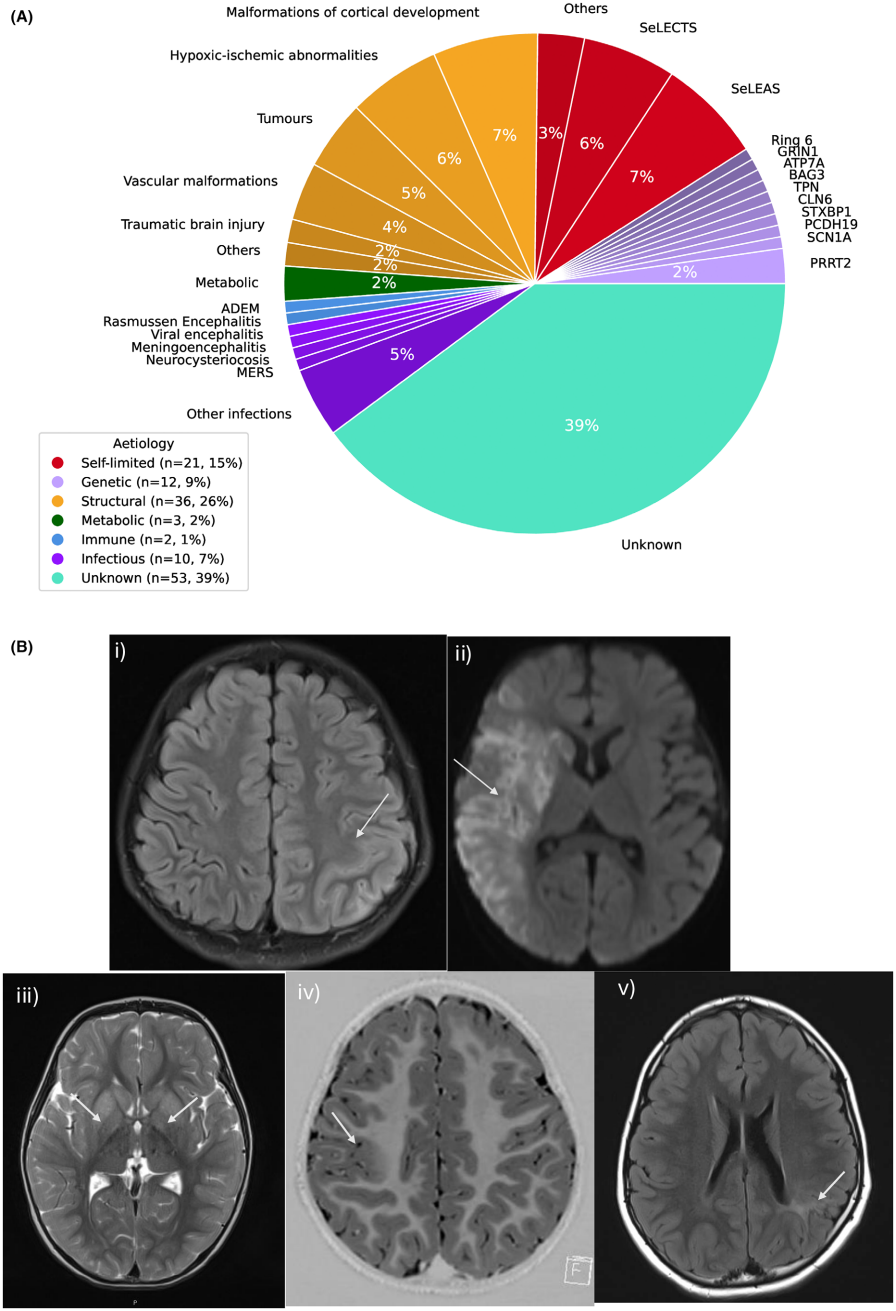
(A) Etiology subgroups. Percentages are 1% or less unless otherwise stated. (B) Case examples of axial neuroimaging findings of new‐onset focal seizures: (i) Left perirolandic and parietal T2 FLAIR hyperintensity s/o focal meningoencephalitis. (ii) Restricted diffusion (DWI) in the right frontal, parietal, temporal lobes, and basal ganglia indicates MCA infarct. (iii) Symmetrical T2 hyperintensity in bilateral lentiform nuclei and head and body of caudate nuclei in propionic acidemia. (iv) Right centroparietal T1 gray‐white matter blurring s/o focal cortical dysplasia. (v) Thinning of left posterior parietal cortical gray matter with T2 FLAIR hyperintensity in adjacent subcortical white matter. DWI, diffusion weighted image; FLAIR, fluid attenuated inversion recovery; S/o, suggestive of.

The most common etiologies were structural (*n =* 36, 26%), self‐limited childhood focal epilepsy (*n =* 21, 15%) and unknown (*n =* 53, 39%). Table 1 and Figure 2A show that amongst the structural etiology, the most prevalent types were malformations of cortical development (*n* = 9, 6% of total cohort) and hypoxic–ischemic abnormalities (*n* = 8, 6%). The most common examples of SeLFEs were self‐limited epilepsy with autonomic seizures (*n* = 9, 6%) and SeLECTS (*n* = 8, 6%). The least common epilepsy etiologies were metabolic (*n* = 3, 2%), genetic (*n* = 12, 9%), and inflammatory (*n =* 12, 9%). Among 38 patients (27%) who underwent genetic testing, gene and chromosome abnormalities included *PRRT2* (most common, *n =* 3), *SCN1A*, *PCDH19*, *STXBP1*, *CLN6*, *TPN*, *BAG3*, *ATP7A*, *GRIN1*, and ring chromosome 6. Within inflammatory causes, examples of immune etiology included acute disseminated encephalomyelitis, Rasmussen encephalitis and infectious etiologies included viral encephalitis, meningoencephalitis, and neurocysticercosis. There were more abnormal findings in EEG (*n* = 115, 82%) (Figure S1) compared to neuroimaging (*n* = 54, 39%) (Figure 2B). EEG findings involved background slowing, asymmetry and attenuation (*n =* 48, 42% of EEG abnormalities), interictal epileptiform discharges (*n* = 83, 72%), and ictal events captured (*n* = 19, 17%).

The mean follow‐up duration of patients who received ASMs on discharge was 2.8 years, of whom 41 (32%) were drug resistant. This included one patient with acute lymphoblastic leukemia and SeLECTS etiology that evolved into epileptic encephalopathy with spike‐wake activation in sleep. Eight patients (6%) received epilepsy surgery, of which temporal lobe resection was the most common, and six were seizure‐free afterwards. None of these patients received neuromodulation. Amongst the epilepsy surgery group, four patients presented with tumors, three with malformations of cortical development, and one with Rasmussen encephalitis. Patients with inflammatory etiology were also administered immunomodulatory treatment such as oral steroids (*n* = 5, 4%), intravenous steroids (*n* = 3, 2%), and intravenous immunoglobulin (*n* = 1, 1%). One patient received ketogenic diet as a nonpharmacological treatment.

### 3.2 Clinical associations for seizure etiology

Table 2A shows significant differences between self‐limited, known and unknown etiologies for proportions of explosive onset seizures (*p* = 0.02), age of seizure onset (*p =* 0.01), abnormal neuroimaging findings (*p <* 0.001) and drug resistance (*p* < 0.001). In particular, patients with known etiologies had higher proportions of explosive onset seizures (*n* = 28, 44%), earlier age of seizure onset (median 2.6 years), abnormal neuroimaging findings (*n* = 43, 68%), and drug resistance (*n* = 25, 40%). The differences in clinical characteristics were also seen in individual known etiologies, as shown in Table 2B. Explosive onset seizures were predominantly present in metabolic (*n* = 2, 67%), inflammatory (*n* = 7, 58%) and genetic (*n* = 6, 50%) etiologies. Focal neurological deficits were more commonly seen in metabolic (*n =* 1, 50%) or structural (*n* = 8, 22%) etiologies. Children with metabolic and genetic etiologies showed the earliest median age of seizure onset at 0.5 years and 0.9 years, respectively. As shown in Table S2, infants accounted for the majority of genetic etiologies (*n* = 7, 58% of genetic) and metabolic etiologies (*n* = 2, 67% of metabolic). Children aged 1–4 years represented the majority of the remaining etiologies. The majority of children in the 9–12 and 13–17 age groups were unknown etiologies.

**TABLE 2:** Clinical associations for etiology in children with new‐onset focal seizures. (A) Broad etiology group analysis (known, self‐limited, and unknown groups). (B) Sub‐group analysis (genetic, structural, metabolic, inflammatory, self‐limited, and unknown groups).

(A)				
Characteristics	Self‐limited (*n* = 21)	Known (*n* = 63)	Unknown (*n* = 53)	*p* value
Mode of onset				0.02
Explosive	3	28	14	
Nonexplosive	18	35	39	
Focal neurological abnormalities				0.1
Yes	2	10	2	
No	19	53	51	
Age of seizure onset				0.01
Median (years)	4.9	2.6	5.6	
MRI/CT				<0.001
Abnormal	3	43	8	
Normal/Not done	18	20	45	
Drug responsiveness				<0.001
Resistant	1	25	9	
Responsive	18	33	31	

(B)							
Characteristics	Self‐limited (*n* = 21)	Genetic (*n* = 12)	Structural (*n* = 36)	Metabolic (*n* = 3)	Inflammatory (*n* = 12)	Unknown (*n* = 53)	*p* value
Mode of onset							0.04
Explosive	3	6	13	2	7	14	
Nonexplosive	18	6	23	1	5	39	
Focal neurological abnormalities							0.04
Yes	2	0	8	1	1	2	
No	19	12	28	2	11	51	
Age of seizure onset							0.01
Median (years)	4.9	0.9	3.6	0.5	5.0	5.6	
MRI/CT							<0.001
Abnormal	3	2	32	2	8	8	
Normal/Not done	18	10	4	1	4	45	
Drug responsiveness							<0.001
Resistant	1	7	19		5	9	
Responsive	18	5	16		6	31	

**FIGURE 3:**
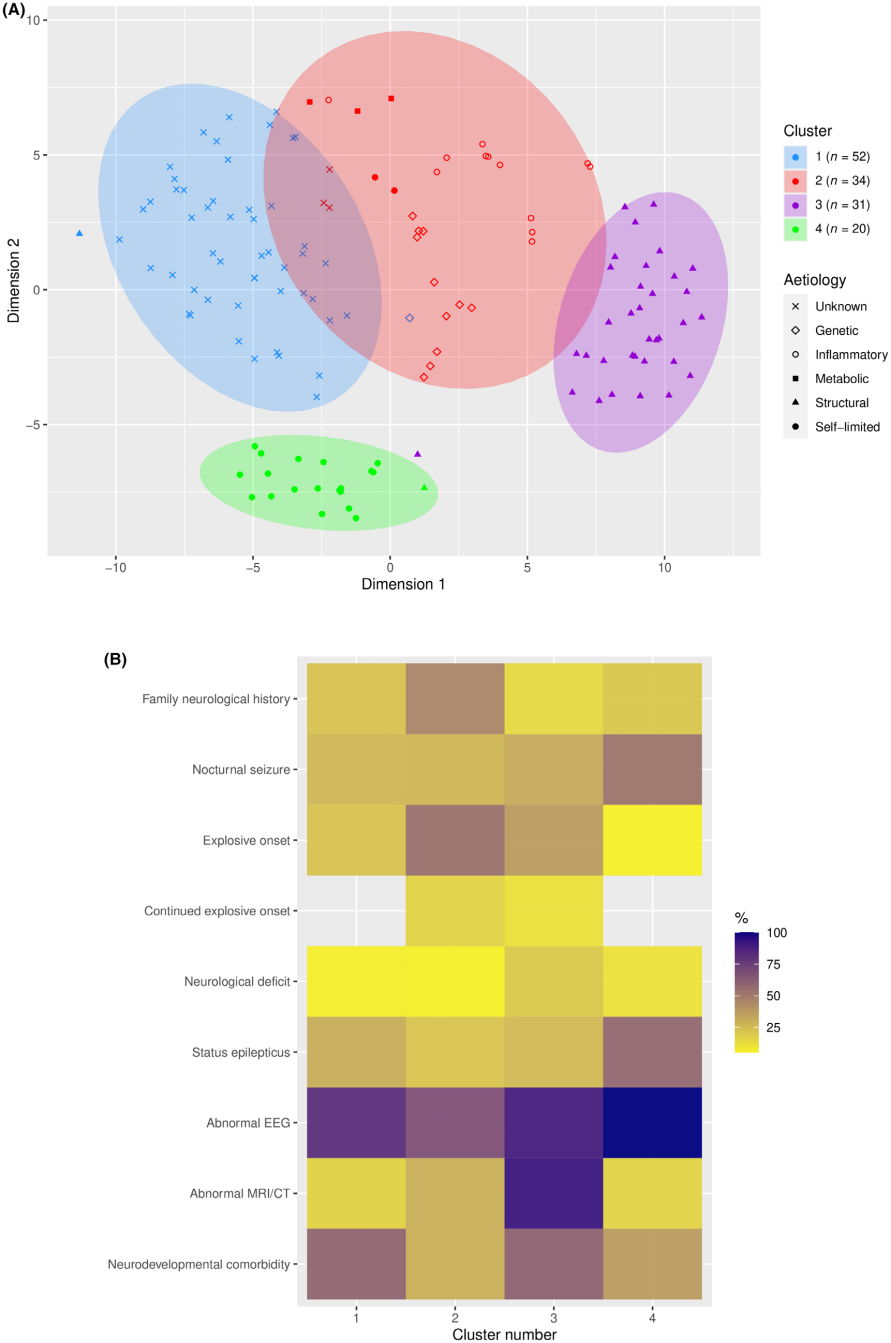
Hierarchical cluster analysis map showing clinical associations for children with new‐onset focal seizures. Predominant etiology of patients in clusters: 1 = unknown (*n* = 52), 2 = genetic/inflammatory/metabolic (*n* = 34), 3 = structural (*n =* 31), 4 = self‐limited (*n* = 20). (A) T‐distributed stochastic neighbor embedding of four clusters. (B) Proportion‐based heatmap of clinical characteristics in each cluster.

Hierarchical cluster analysis grouped the patients into four clusters, as shown in Figure 3A. Figure 3B displays the similarities and differences in clinical characteristics among the four clusters. Cluster 1 (*n* = 52) consisted of patients with unknown etiologies (96% of the cluster), which showed a higher percentage of abnormal EEG and neurodevelopmental comorbidities with relatively limited explosive seizures/neuroimaging abnormalities. In contrast, Cluster 2 (*n =* 34) involved combined patients with genetic, inflammatory, and metabolic etiologies and showed a higher association with explosive onset seizures, family history, and abnormal EEG. Structural etiologies were prevalent in Cluster 3 (*n* = 34, 100% of cluster) and showed a higher proportion of abnormal neuroimaging findings and EEG, neurological deficit and neurodevelopmental comorbidities. The SeLFEs represented 95% of Cluster 4 (*n* = 20) with a higher proportion of abnormal EEGs, nocturnal seizures, prolonged seizures, and normal neuroimaging. Patients with explosive onset or continued explosive seizures were more likely to be grouped in clusters with known etiology including genetic, metabolic, inflammatory, and structural. The case examples of neuroimaging and EEG abnormalities have been described in Figure 2B and Figure S1, respectively.

### 3.3 Anti‐seizure medications and predictors of drug resistance

One hundred twenty‐nine patients (92%) were prescribed ASMs, with a median of two medications from first admission to last follow‐up. Figure S2a shows the most common medications were levetiracetam followed by carbamazepine. Clobazam and valproate were often used as second‐line treatments. Uncommon ASMs included cannabidiol, rufinamide, and brivaracetam. Figure S2b represents the range of associations between ASMs that were prescribed to patients. In particular, a high degree of association is shown between levetiracetam, clobazam, carbamazepine, and valproate.

Table 2 shows that a significant proportion of patients with specific etiologies (61% of structural, 58% of genetic, and 45% of inflammatory) showed resistance to anti‐seizure medications. By contrast, 23% of unknown and 5% of SeLFE etiologies led to drug resistance. As described in Table S3, multivariable risk factors for drug resistance were genetic (OR 6.7; 95% CI 1.6–31.8, *p* = 0.01), structural (OR 6.4; 95% CI 2.3–19.5, *p* < 0.001), and inflammatory (OR 4.6; 95% CI 1.0–21.2, *p* = 0.05) etiologies.

## 4 DISCUSSION

This study analyzed the clinical features and outcomes of pediatric patients with new‐onset focal seizures. We extend previous literature that described all new‐onset seizure types, which can have different etiologies and treatment implications compared to focal seizures.^22^ A greater proportion of the cohort developed epilepsy compared to previous studies investigating new‐onset seizures in children or adult populations (98% vs. 60%–76%).^9^, ^23^, ^24^, ^25^ This can be due to the inclusion of only focal seizures in the current study, which account for the majority of newly diagnosed epilepsy.^2^, ^26^ Although the diagnosis of focal seizures was determined by neurologists or pediatricians' documented assessments, some cases may have been missed in our study due to incomplete histories or unwitnessed focal seizures that evolve early into generalized tonic–clonic seizures.

We reported various etiologies amongst patients, with an increased proportion of known etiologies (61%) compared to previous studies on new‐onset focal seizures/epilepsy in children (44%–50%).^27^, ^28^ This may be due to recent diagnostic advances and availability in genetic, neuroimaging, and immunological testing. Structural etiologies accounted for the highest proportion of known etiologies, which were consistent with previous studies.^9^, ^23^, ^26^, ^27^

SeLFEs have had a prevalence of 22% similar to the reported 16%–23% of all pediatric epilepsies.^3^, ^11^ Although previous studies have shown a positive family history of epilepsy and presumed polygenic complex inheritance in SeLFE cases, we did not include these cases under genetic etiology due to a lack of monogenic or chromosomal cause.^19^ This has led to a lower prevalence of genetic causes compared to a recent study that showed 33% of patients with genetic etiology, which included presumptions of genetic etiology from family history and epilepsy syndrome.^3^ Genetic etiologies may also be underestimated, as only a portion of children with new‐onset focal seizures underwent genetic testing. Furthermore, without brain tissue analysis, blood‐based genetic testing may overlook somatic variants in the GATOR‐mTORC1 pathway that can lead to cortical malformations.^29^

Amongst the inflammatory, genetic and metabolic etiologies, there was a significant proportion of explosive onset of seizures. Recent studies evaluating etiologies of seizures have not investigated their association with explosive onset despite the occurrence of this onset in 38% of pediatric patients.^24^ Small case studies have shown that explosive onset has been common in specific inflammatory etiologies, including febrile infection‐related epilepsy syndrome and anti‐NMDA receptor encephalitis, and structural etiologies such as focal cortical dysplasia.^24^, ^30^, ^31^ Furthermore, examples of genetic etiologies, such as *PRRT2* pathological variants, have been associated with an initial explosive onset of seizures.^32^, ^33^ Hence, it is important to consider broader etiologies in children presenting with explosive onset seizures for appropriate patient management. It is also important to acknowledge that ongoing seizures and the development of epilepsy can be due to the sequelae of encephalitis that are caused by injury to the cortical or mesial temporal regions. Focal neurological deficits were more frequent in patients with structural etiologies compared to others possibly due to ictal dysfunction. Previous studies have shown that hypoxic–ischemic structural abnormalities can precipitate certain focal deficits such as Todd's paresis via COX‐2‐dependent hypoperfusion.^34^, ^35^, ^36^

The proportion of abnormalities in neuroimaging findings among pediatric seizures also varied in the literature (15%–57%) and the current study (39%).^9^, ^22^, ^24^, ^27^, ^28^, ^37^, ^38^, ^39^ Structural etiologies also have a significantly higher proportion of abnormal neuroimaging findings, which highlight the importance of neuroimaging in detecting subtle lesions.^4^, ^38^ Hypoxic–ischemic abnormalities and malformations of cortical development were the most prevalent structural abnormalities, similar to previous literature.^3^, ^27^, ^40^ Compared to neuroimaging abnormalities, abnormal findings in EEG were more common at 82% but were not associated with different etiologies. Previous studies have shown that there is a range of prevalence for abnormal EEG findings (22%–89%).^3^, ^9^, ^22^, ^24^, ^25^, ^27^, ^37^, ^38^, ^39^ This can be due to a combination of differences in methodology such as seizure onset, age of onset, proportion and timing of patients receiving the investigation, and geographical location.

Neurodevelopmental issues are significant comorbidities in children presenting with new‐onset focal seizures, and have varied from 10% to 55% in pediatric epilepsies.^9^, ^18^, ^24^, ^26^, ^28^, ^38^, ^41^ In particular, language/learning difficulties and autism spectrum disorders were seen, likely due to the seizures originating from mesial structures that affect executive function and memory, as discussed in previous studies.^24^, ^38^ A study investigating psychiatric comorbidities in children with new‐onset epilepsy also showed that after excluding predominant neurodevelopmental disorders, there were significantly higher rates of depressive and anxiety disorders compared to a nonepileptic comparison group.^18^ The lower rate of mood disorders in our study is possibly due to a younger cohort (mean age 5.2 years vs. 12.7 years) and associated screening difficulties.^42^

In our study, the age of seizure onset showed a significant association with the etiology of seizures. This is supported by previous studies showing the prevalence of infants in genetic etiologies, and later onset for structural and other etiologies.^2^, ^40^

**FIGURE 4:**
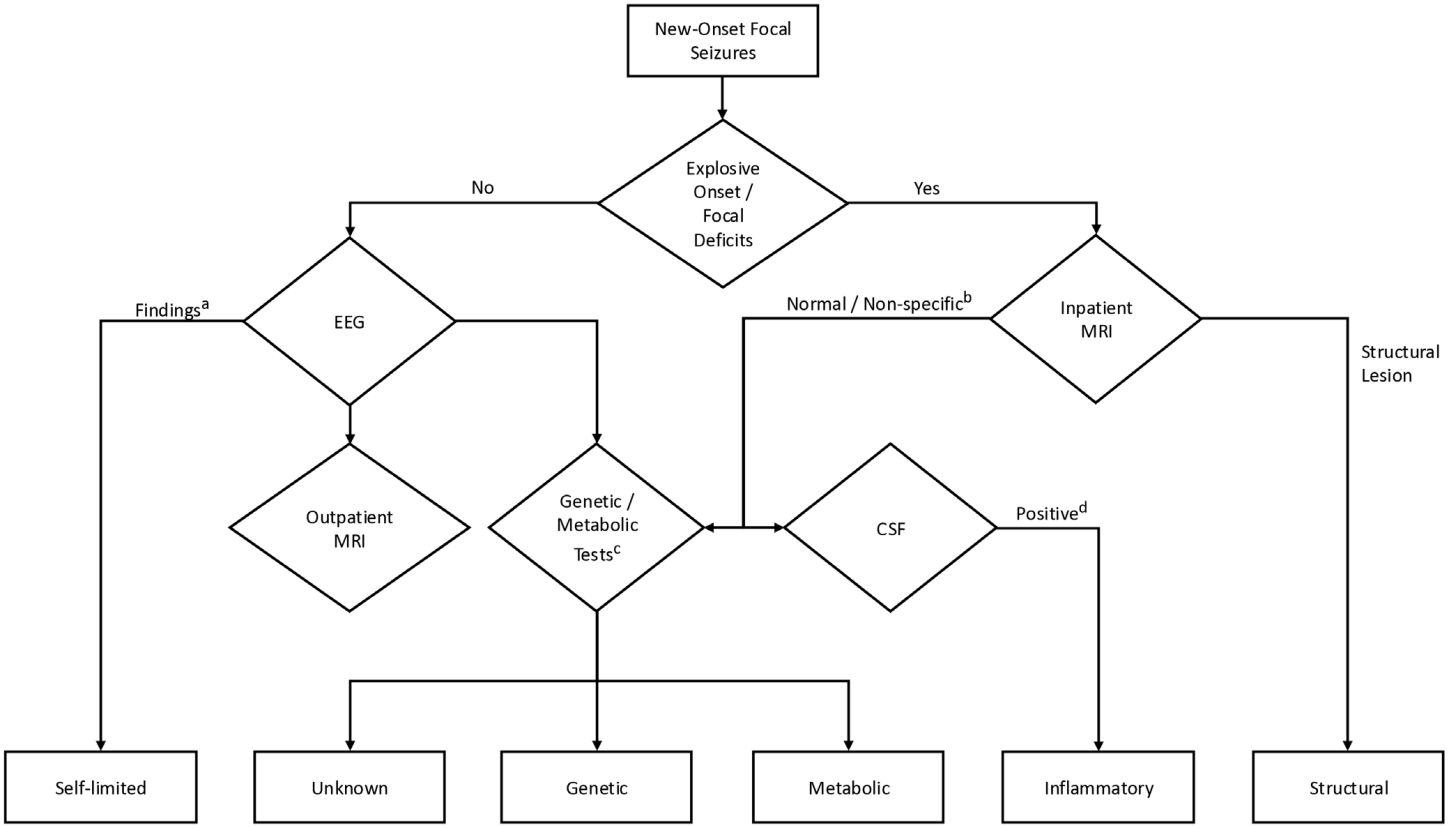
Algorithm for etiology in new‐onset focal seizures in children. ^a^EEG findings described with self‐limited childhood focal epilepsy including self‐limited epilepsy with centrotemporal spikes, multifocal high amplitude, or occipital spikes. ^b^Other neuroimaging may be shown in nonstructural etiologies. Further investigations rely on clinical context. ^c^Genetic tests include CGH Microarray/Epilepsy gene panel showing pathogenic variants. In resource‐rich settings, whole exome sequencing or whole genome sequencing can be considered for higher yield. Metabolic tests include blood, urine, and CSF. ^d^Inflammatory CSF findings include antibodies and pleocytosis (>5 NC/μL).

Based on the findings from the study and literature review (Table S1), the study proposes an algorithmic approach to etiologies in children with new‐onset seizures, shown in Figure 4. Patients with explosive onset seizures or focal neurological deficits should undergo MRI as inpatients, which can determine a structural etiology via structural lesions, or inflammatory, genetic, or metabolic etiologies otherwise. Further analysis using cerebrospinal fluid (CSF), genetic and metabolic testing will help determine the underlying etiology for appropriate management. In the absence of explosive onset or focal neurological abnormalities, EEG as an initial investigation can identify specific groups of SeLFEs, such as SeLECTS, and may guide the need for further investigations including outpatient MRI and genetic testing in an unknown group.^9^, ^22^, ^24^, ^25^, ^26^, ^27^, ^37^, ^38^, ^39^ The triage in inpatient and outpatient investigations in our proposed approach can lead to appropriate resource allocation and minimize the length of hospital stay.

Furthermore, it is important to consider patients who develop drug resistance, as they constitute a noticeable proportion of the cohort, with 32% in the current study and 17%–36% in previous studies.^9^, ^11^, ^26^, ^27^, ^28^, ^31^ The current study revealed that structural, genetic, and inflammatory etiologies increased the risk of drug resistance, which is consistent with previous studies that show higher susceptibility in patients with known etiologies.^14^, ^26^, ^27^, ^43^ Similar to a recent study investigating focal and generalized epilepsy, structural factors were the most prevalent known etiology in drug‐resistant patients.^40^ However, other risk factors described in the literature were inconsistent with our analysis, such as the presence of younger age, neurodevelopmental comorbidities, a history of febrile seizures and abnormal neuroimaging/EEG findings.^14^, ^26^ This could be due to differences in methodology, such as age stratification and setting a year‐minimum period for seizure freedom in other studies.

As known etiologies can increase the risk of drug resistance; the use of etiology‐driven treatment should be recognized in improving patient outcomes, particularly in medical and psychiatric comorbidities.^16^, ^17^, ^18^ Examples of etiology‐driven treatment include combined surgery and medical therapy in structural etiology, early immunomodulation in inflammatory etiology, removing contraindicated medications in patients with SCN1A‐related seizures, and ketogenic diet in metabolic etiologies such as glucose transporter deficiency.^8^, ^44^, ^45^, ^46^ In contrast to effective etiology‐driven treatment, the optimal choice of anti‐seizure medications has not been established for drug‐resistant epilepsy, and is consistent with the range of associations between ASMs in our study.^47^ It is suggested that ASMs with different mechanisms of action, and those with synergistic effects like valproate and lamotrigine, can improve seizure treatment.^47^

The study was limited to a single‐center cohort with retrospective analysis, which may not be generalized to the global pediatric population with focal seizures. Some patients also had incomplete follow‐up data due to relocation or lack of continued care, which may lead to an overestimated proportion of late comorbidities or drug resistance due to reduced medical access from healthy patients.^48^ Despite the limitations, the study addresses the importance of explosive onset in seizures for determining etiologies, which can further lead to optimized treatment for patients. Future studies can investigate large multicenter cohorts to validate etiology and treatment outcomes.

## 5 CONCLUSIONS

In children presenting with new‐onset focal seizures, we highlight the important associations between etiologies and explosive seizure onset, age, neurological deficits, and abnormal neuroimaging. By considering known etiologies as risk factors for drug resistance, the study promotes the need for improved monitoring and specific treatment for these patients. We also suggest an investigation algorithm that can aid clinicians in appropriate seizure management.

## AUTHOR CONTRIBUTIONS

B.C.L. designed the study, performed a literature review, collected data, analyzed it, and drafted the paper under the supervision of K.K. E.H.B. reviewed the statistical analysis. R.C.D./S.S.M./S.G./C.W./D.G. provided part of the clinical data. All the above authors discussed the methodology, presentation of the data, and edited the paper.

## FUNDING INFORMATION

This work was supported by a scholarship from the National Health and Medical Research Council EL1 Investigator Grant 2017279.

## CONFLICT OF INTEREST STATEMENT

All authors report no disclosures. We confirm that we have read the Journal's position on issues involved in ethical publication and affirm that this report is consistent with those guidelines.

## Supporting information


Figure S1.

Figure S1b.

Figure S1c.

Figure S1d.

Figure S2a.

Figure S2b.

Tables S1–S3.
