# Supplementary figures and images for: Predictors of etiology and drug resistance in children with new‐onset focal seizures
Source: Epilepsia Open. 2026 Jan 16;11(1):123–35. doi: 10.1002/epi4.70179 (PMC12903812; doi:10.1002/epi4.70179)

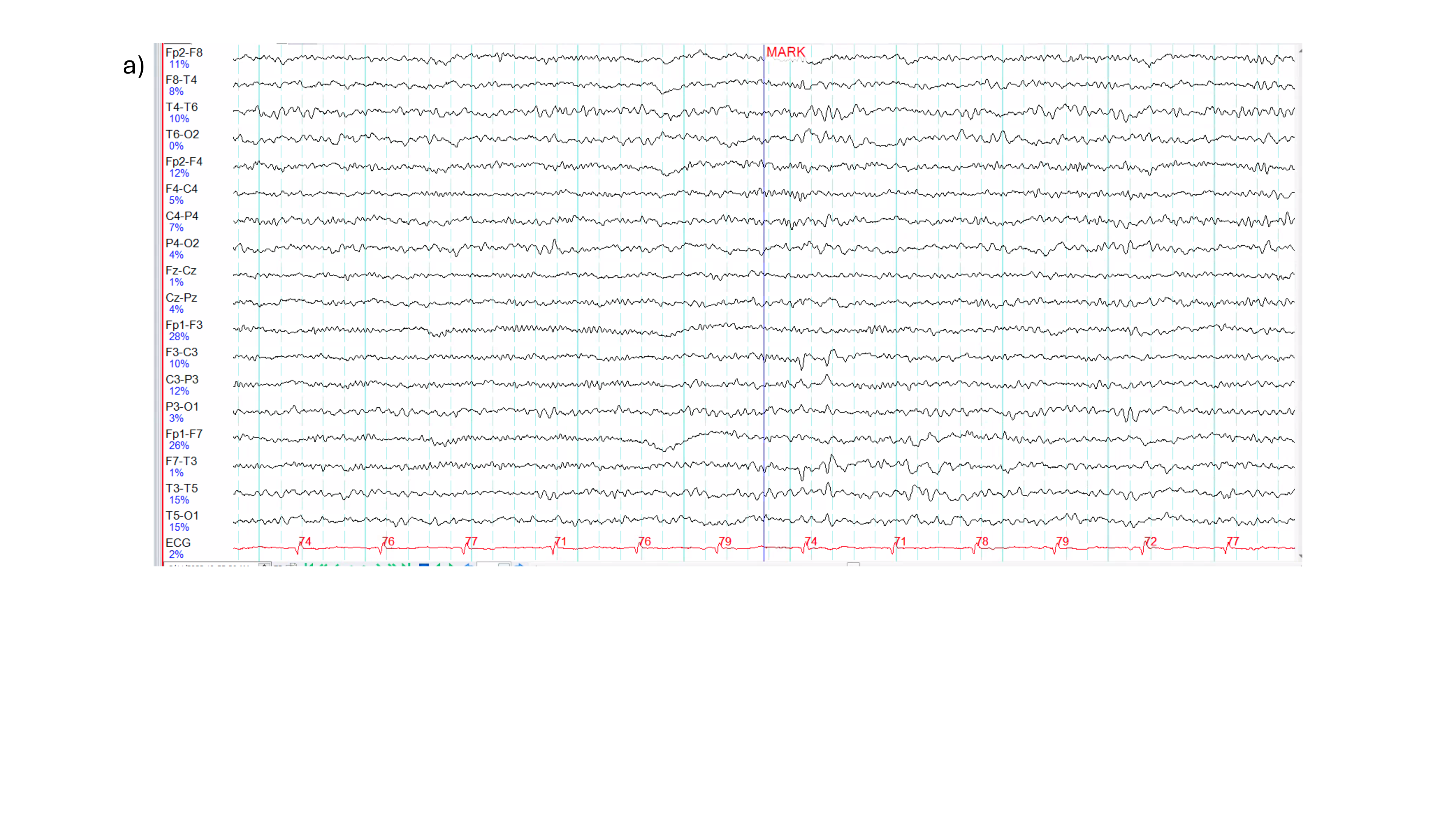

Supplement: Supplementary file 1 — Figure S1. [file EPI4-11-123-s007.tiff]

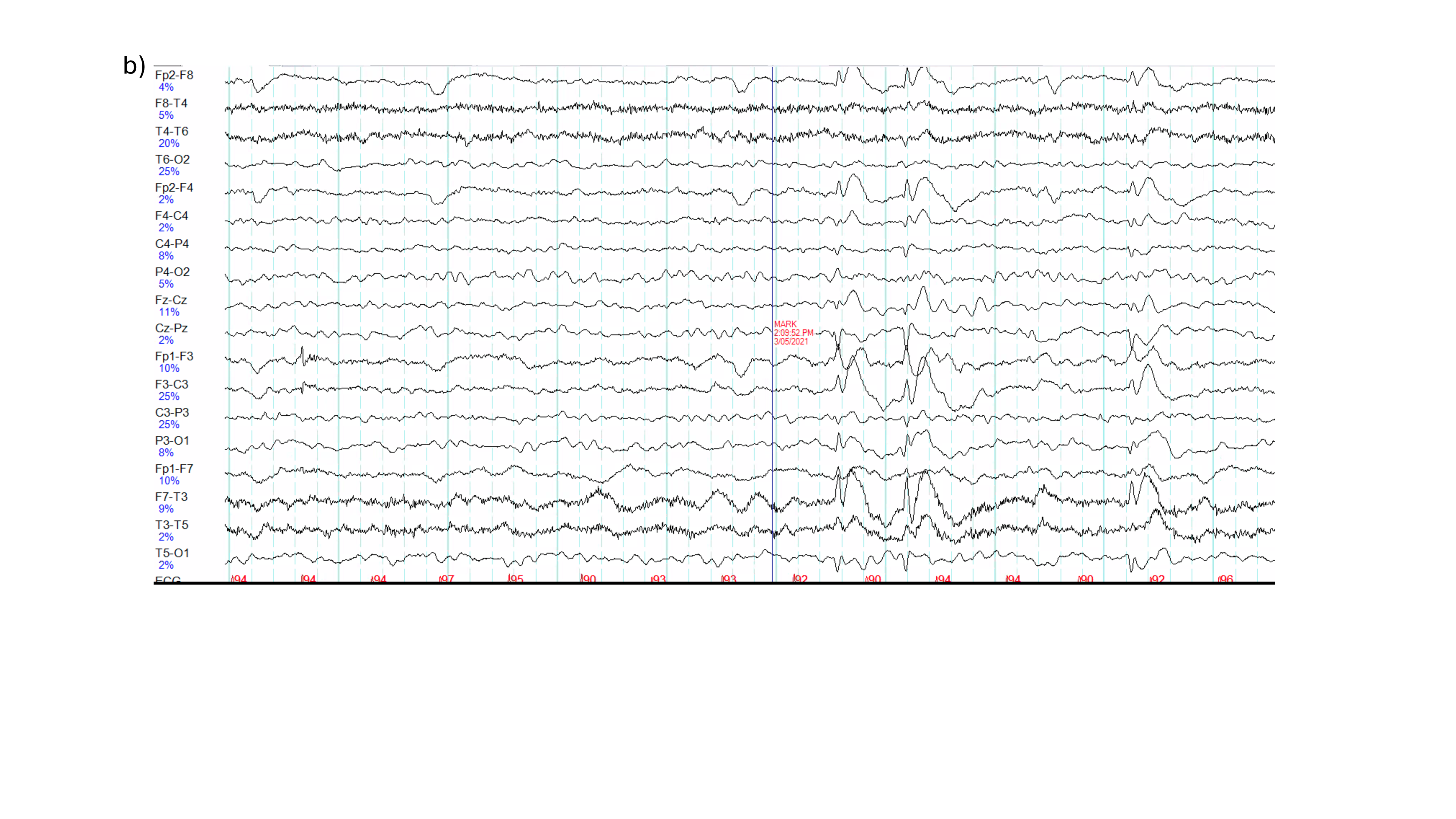

Supplement: Supplementary file 2 — Figure S1b. [file EPI4-11-123-s003.tiff]

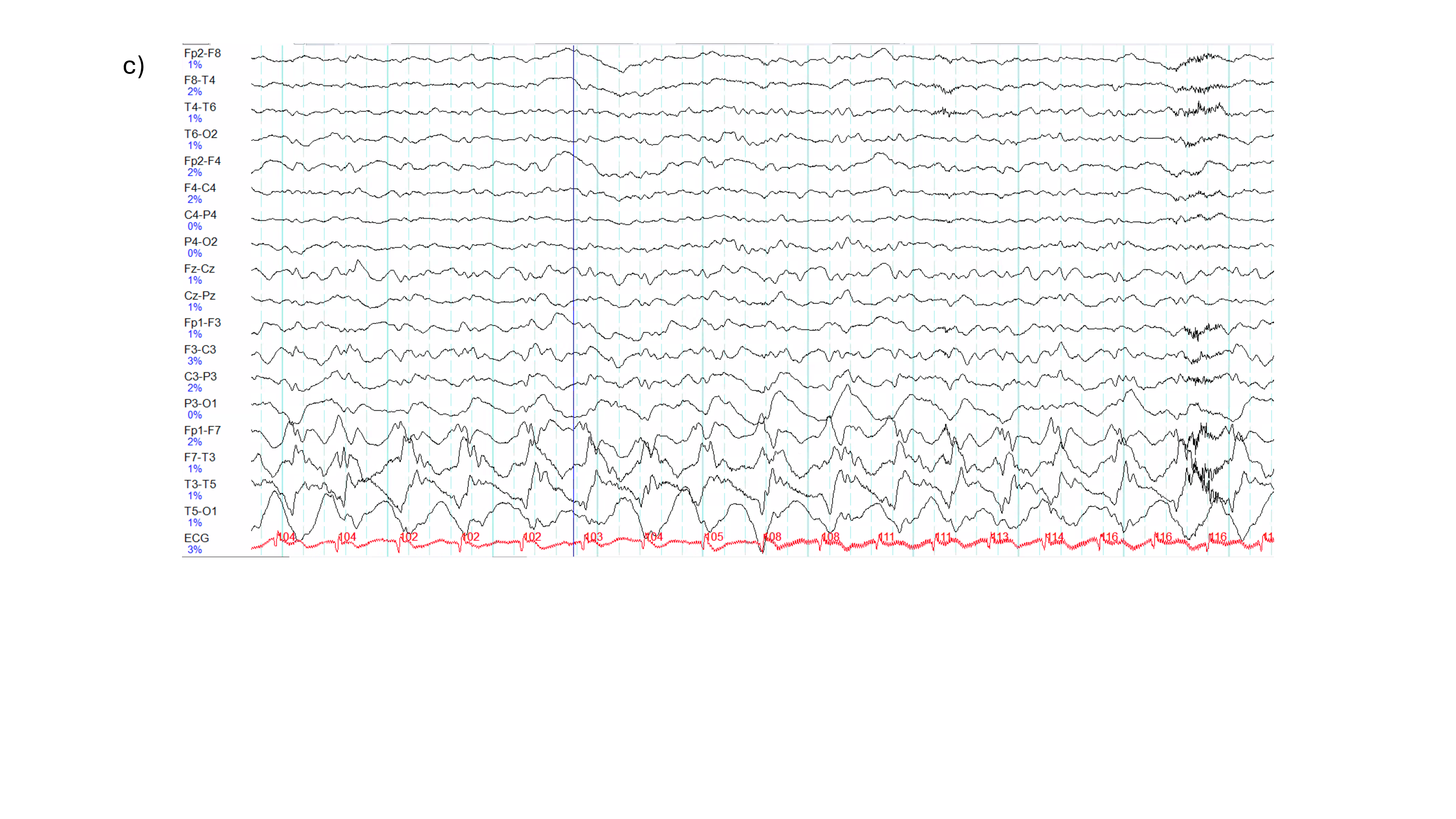

Supplement: Supplementary file 3 — Figure S1c. [file EPI4-11-123-s002.tiff]

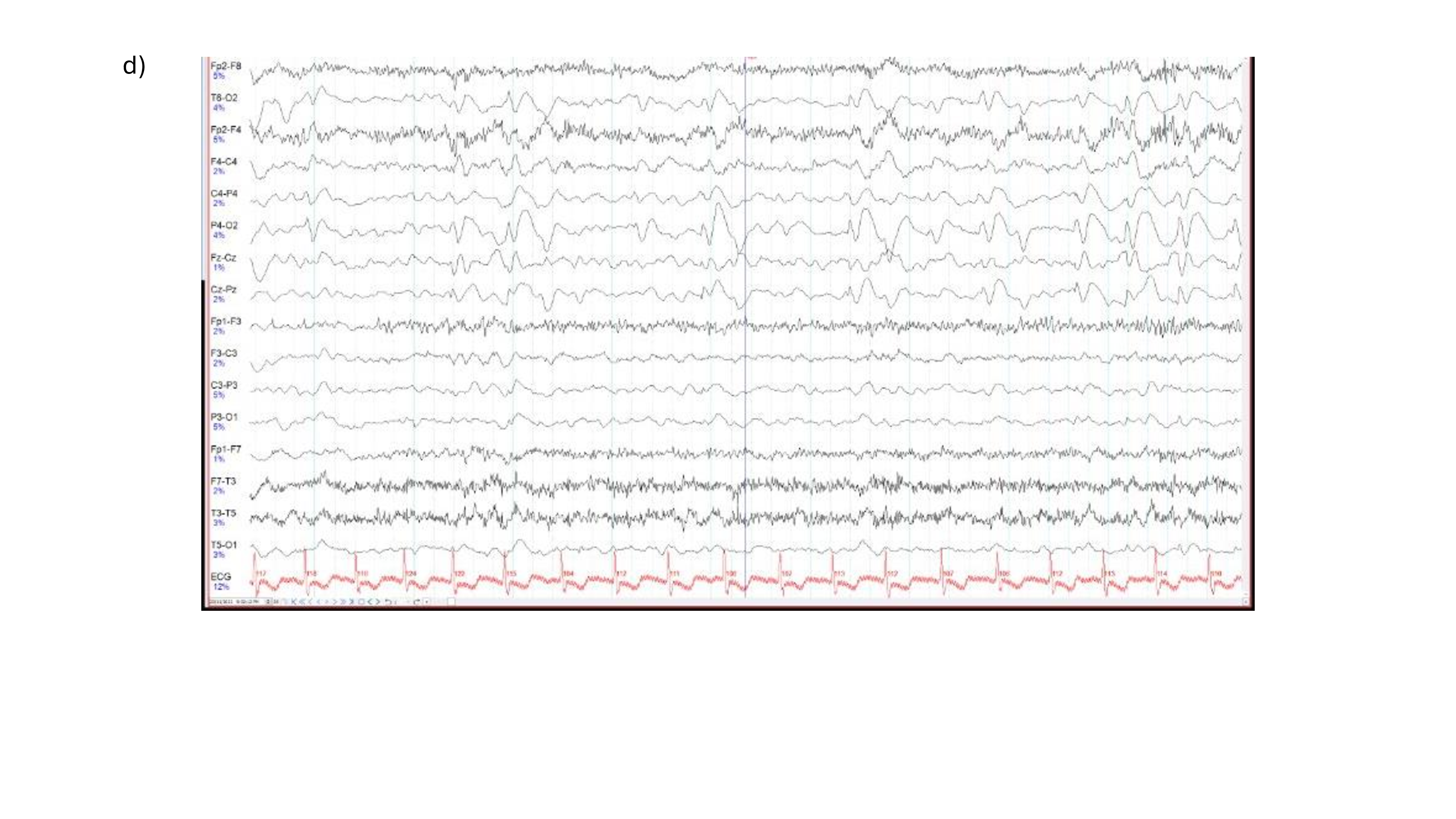

Supplement: Supplementary file 4 — Figure S1d. [file EPI4-11-123-s004.tiff]

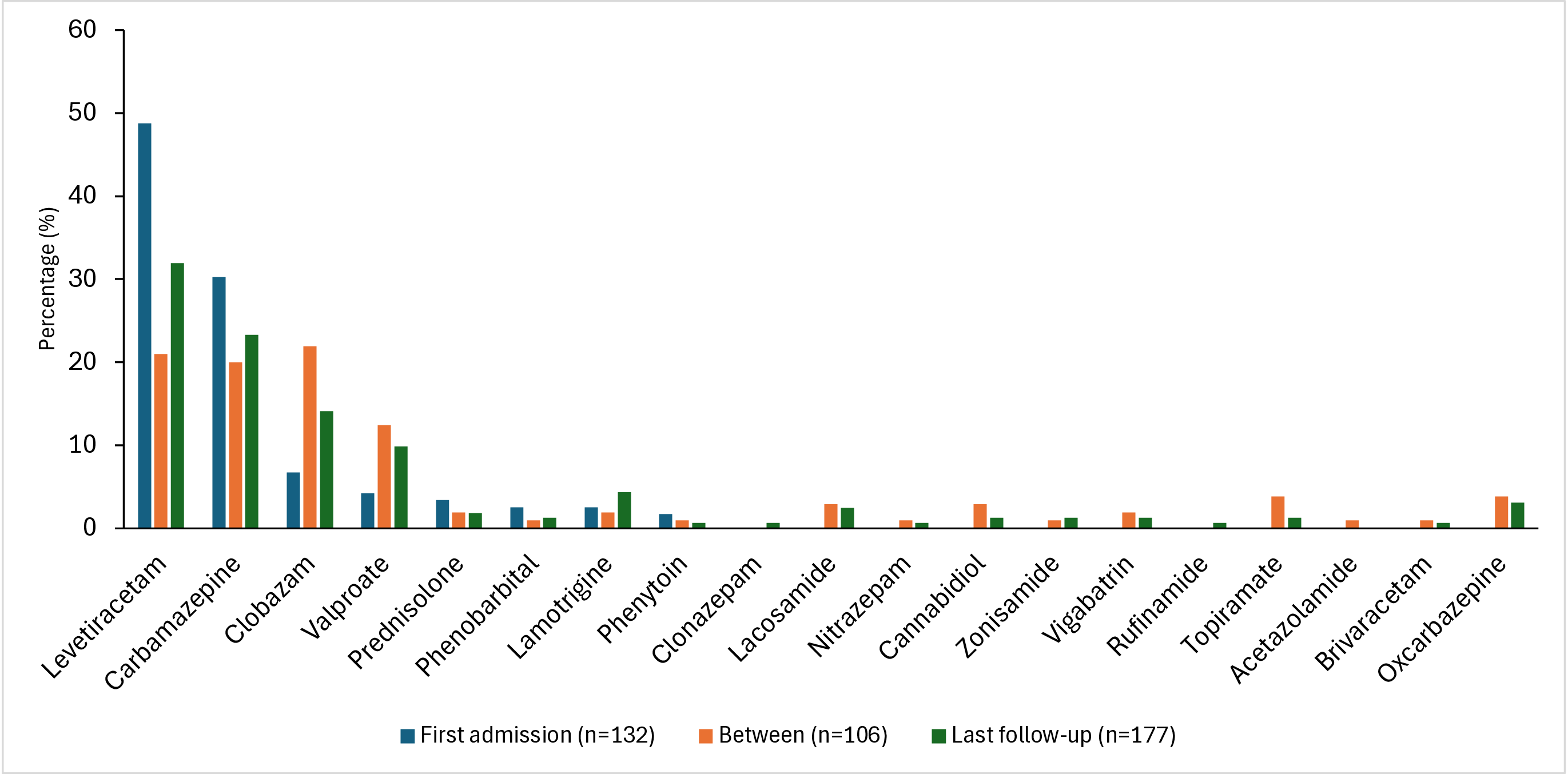

Supplement: Supplementary file 5 — Figure S2a. [file EPI4-11-123-s001.tiff]

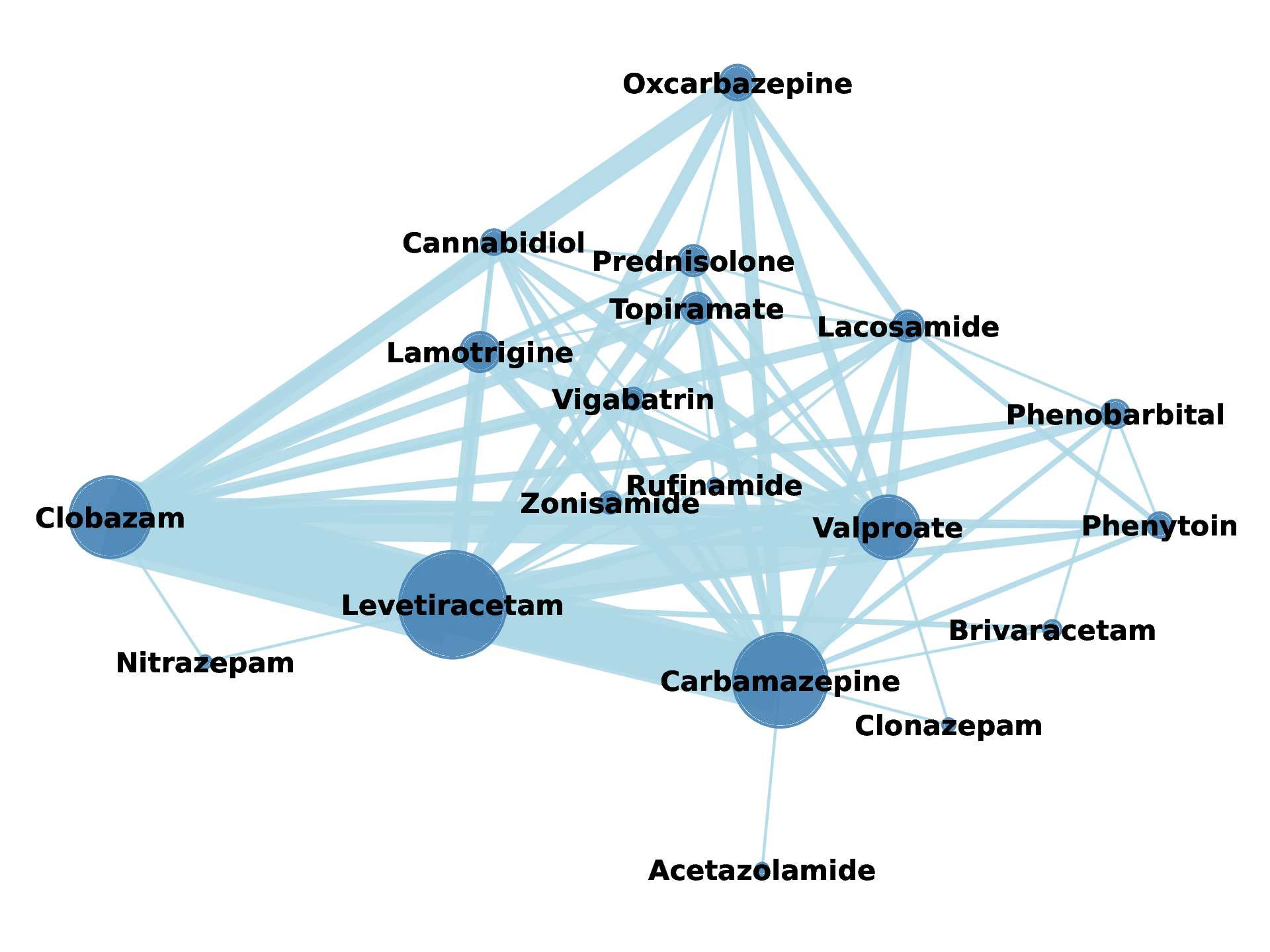

Supplement: Supplementary file 6 — Figure S2b. [file EPI4-11-123-s005.tiff]
