# Supplementary material for: Predictors of etiology and drug resistance in children with new‐onset focal seizures
Source: Epilepsia Open. 2026 Jan 16;11(1):123–35. doi: 10.1002/epi4.70179 (PMC12903812; doi:10.1002/epi4.70179)
Supplement: Supplementary file 7 — Tables S1–S3. [file EPI4-11-123-s006.docx]

**Table S1.** Literature review of the aetiology, comorbidities and outcomes in children with seizures.

| **Authors** | **Year** | **No. of Paediatric Patients** | **No. of New-onset Focal Seizures (%)** | **Mode of onset (%)** | **Median Age of Seizure Onset (years)** | **Etiology (%) of all seizure types** | **% neuro-comorbidities** | **% abnormal EEG** | **% abnormal MRI** | **% abnormal CT** | **% epilepsy** | **% drug resistance** | **First-line AED (%)** |  |
| --- | --- | --- | --- | --- | --- | --- | --- | --- | --- | --- | --- | --- | --- | --- |
| Vikin, et al.^1^ | 2025 | 1053 | 639 (61) | N/A | 6 | Structural (21), Genetic (33), Infectious (1), Metabolic (1), Immune (0.2), Unknown (47) | N/A | 89 | 32 | N/A | 100 | N/A | N/A |  |
| Zuo, et al.^2^ | 2024 | 167 | 53 (50) | N/A | 3 | Structural (23), Genetic (19), Immune (4), Unknown (47) | N/A | N/A | N/A | N/A | 100 | 100 | N/A |  |
| Itamura, et al.^3^ | 2023 | 281 | N/A | N/A | 7 (mean) | Unknown (91), Structural (4), Genetic (3), Infectious (1), Metabolic (0.4) | N/A | N/A | N/A | N/A | 100 | 18 | N/A |  |
| Poke, et al.^4^ | 2023 | 235 | N/A | N/A | 1.28 | N/A | N/A | N/A | N/A | N/A | N/A | N/A | N/A |  |
| Ali, et al.^5^ | 2022 | 201 | 49 (24) | N/A | <1 (41%), 1-5 (19%), 5-10 (25%), 10-18 (28%) | N/A | N/A | 53 | 36 | | N/A | N/A | N/A |  |
|  |  |  |  |  |  |  |  |  |  |  |  |  |  |  |
| Shi, et al.^6^ | 2022 | 58 | N/A | N/A | 23 | Immune (15), Unknown (85) | N/A | 78 | 8 | N/A | N/A | N/A | N/A |  |
| Egesa, et al.^7^ | 2022 | 256 | N/A | N/A | 1.6 | Unknown (12), Infectious (43), Structural (45) | 55 | 60 | 51 | N/A | 100 | N/A | N/A |  |
| Kim, et al.^8^ | 2020 | 492 | 97 (20) | Explosive (38) | 7.2 (average) | N/A | 28 | 51 | 15 | 5 | 76 | N/A | levetiracetam (39) |  |
| Roy, et al.^9^ | 2020 | 6 | 6 (100) | Explosive (100) | > 12 | Autoimmune | 0 | 100 | 0 | N/A | 100 | 17 | methylprednisolone and AEDs |  |
| Wirrell, et al.^10^ | 2014 | 468 | 468 (100) | N/A | 5.4 | Unknown (56), Structural/Metabolic (40), Genetic (4) | 42 | 66 | 32 | N/A | 100 | 36 | N/A |  |
| Suleiman, et al.^11^ | 2013 | 114 | 56 (49) | N/A | 3.4 (antibody-pos), 2 (antibody-neg) | Unknown (40), Structural (22), Infections/inflammation (13), Metabolic (5), Genetic (3), Other (17) | 32 | 76 | 50 | N/A | 74 | 27 | N/A |  |
| Wirrell, et al.^12^ | 2012 | 127 | 84 (66) | N/A | 1.1 | Unknown (43), Genetic (18), Structural/metabolic (39) | 52 | 69 | 33 | N/A | 100 | 35 | N/A |  |
| Sierra-Marcos, et al.^13^ | 2011 | N/A | NA | N/A | 52.42 (medium) | Unknown (50), Toxic-metabolic (20), Cerebral chronic lesions (10), Systemic disorders or fever (10), Acute lesions (8), Sleep deprivation (2) | N/A | 62 | 39 | 37 | 60 | N/A | N/A |  |
| Wirrell, et al.^14^ | 2011 | 359 | 244 (68) | N/A | 5.3 | Unknown (50), Structural/metabolic (28), Genetic (22) | N/A | N/A | N/A | N/A | 100 | N/A | N/A |  |
| Hsieh, et al.^15^ | 2010 | 317 | 154 (49) | N/A | <0.5 (38), 0.5-1 (27), 1-2 (34) | N/A | 8 | 50 | 57 | 35 | >71 | N/A | N/A |  |
| Chen, et al.^16^ | 2010 | 319 | 16 (5) | N/A | 2 | Acute symptomatic (6), Febrile (62) | 3 | 22 | 26 | | 4 | N/A | acute benzodiazepine (20) |  |
| Geerts, et al.^17^ | 2010 | 494 | N/A | N/A | 5.5 (mean) | Idiopathic (51), remote sympotmatic (28), Cryptogenic (21) | N/A | N/A | N/A | N/A | 100 | 9 | N/A |  |
| Bhise, et al. | 2009 | 57 | 34 (60) | N/A | 10.1 (mean) | N/A | N/A | N/A | 4 | N/A | 100 | N/A | N/A |  |
| Jones, et al.^18^ | 2007 | 53 | 30 (57) | N/A | 11.5 (mean) | N/A | depression (23), anxiety (36), psychotic (2), ADHD (26), Oppositional defiant (13), Conduct (4), Tic (9) | N/A | N/A | N/A | 100 | N/A | N/A |  |
| Kim, et al.^8^ | 2006 | N/A | N/A | N/A | 24.3 | N/A | 18 | 44 | N/A | N/A | N/A | N/A | valproate (43) |  |
| Doescher, et al.^19^ | 2006 | 181 | 110 (61) | N/A | 9.4 (mean) | N/A | N/A | 72 | 33 | N/A | N/A | N/A | N/A |  |
| Wheless, et al.^20^ | 2004 | 119 | 67 (56) | N/A | 12.8 (mean) | N/A | N/A | N/A | N/A | N/A | 100 | 52 | topiramate (65), carbamazepine (19), valproate (16) |  |
| Sztriha, et al.^21^ | 2002 | 30 | 30 (100) | N/A | 6.8 (mean) | Unknown (50), Structural (43), Other (7) | 10 | 100 | 43 |  | 100 | 17 | N/A |  |
| Shinnar, et al.^22^ | 2001 | 407 | 169 (42) | N/A | N/A | Cryptogenic/idiopathic (84), Remote symptomatic (16) | N/A | N/A | N/A | N/A | N/A | N/A | acute benzodiazepine (8) |  |

**Table S2.** Frequency, per age group, of aetiology in children with new-onset focal seizures.

| Age Group | Total (*n=*140) | Self-limited (*n*=21) | Genetic (*n*=12) | Structural (*n*=36) | Metabolic (*n*=3) | Inflammatory (*n=*12) | Unknown (*n*=53) |
| --- | --- | --- | --- | --- | --- | --- | --- |
| First year | 30 | 2 | 7 | 10 | 2 | 2 | 7 |
| 1 – 4 y | 46 | 9 | 3 | 11 | 1 | 4 | 18 |
| 5 – 8 y | 36 | 7 | 1 | 9 | 0 | 4 | 15 |
| 9 – 12 y | 20 | 3 | 1 | 3 | 0 | 1 | 12 |
| 13 – 17 y | 8 | 0 | 0 | 3 | 0 | 1 | 4 |

**Table S3.** Risk factors for developing drug resistance in children with new-onset focal seizures (multivariable analysis).

| **Characteristics** | **OR (95% CI)** | ***p* value** |
| --- | --- | --- |
| Genetic aetiology | 6.7 (1.6 – 31.8) | 0.01 |
| Structural aetiology | 6.4 (2.3 – 19.5) | <0.001 |
| Inflammatory aetiology | 4.6 (1.0 – 21.2) | 0.05 |
| Self-limited focal epilepsy | 0.2 (0.01 – 1.3) | 0.15 |
| Neurodevelopmental disorders | 1.9 (0.8 – 4.8) | 0.14 |
| Explosive onset | 0.4 (0.1 – 1.1) | 0.07 |

**Supplementary References**

1. Vikin T, Lossius MI, Brandlistuen RE, Chin RF, Aaberg KM. Incidence of childhood and youth epilepsy: A population-based prospective cohort study utilizing current International League Against Epilepsy classifications for seizures, syndromes, and etiologies. *Epilepsia.* 2025.

2. Zuo RR, Jin M, Sun SZ. Etiological analysis of 167 cases of drug-resistant epilepsy in children. *Ital J Pediatr.* 2024;**50**(1):50.

3. Itamura S, Sasaki K, Fujii Y, Okano R. Antiseizure medication treatment outcomes in new‐onset pediatric epilepsy. *Pediatrics International.* 2023;**65**(1):e15523.

4. Poke G, Stanley J, Scheffer IE, Sadleir LG. Epidemiology of Developmental and Epileptic Encephalopathy and of Intellectual Disability and Epilepsy in Children. *Neurology.* 2023;**100**(13):e1363-e75.

5. Ali N, Haider S, Mustahsan S, Shaikh M, Raheem A, Soomar SM, et al. Predictors of abnormal electroencephalogram and neuroimaging in children presenting to the emergency department with new-onset afebrile seizures. *BMC Pediatr.* 2022;**22**(1):619.

6. Shi X, Cai W, Zhang X, Pan H, Huang C, Wang S, et al. Early predictors of new-onset immune-related seizures: a preliminary study. *BMC Neurol.* 2022;**22**(1):503.

7. Egesa IJ, Newton CR, Kariuki SM. Evaluation of the International League Against Epilepsy 1981, 1989, and 2017 classifications of seizure semiology and etiology in a population‐based cohort of children and adults with epilepsy. *Epilepsia Open.* 2022;**7**(1):98-109.

8. Kim LG, Johnson TL, Marson AG, Chadwick DW. Prediction of risk of seizure recurrence after a single seizure and early epilepsy: further results from the MESS trial. *The Lancet Neurology.* 2006;**5**(4):317-22.

9. Roy AG, Vinayan KP, Kannoth S. New onset focal seizure clusters in children: Expanding the spectrum of anti NMDAR encephalitis. *Neurology India.* 2020;**68**(6):1374.

10. Wirrell EC, Wong-Kisiel LC, Nickels KC. Seizure outcome after AED failure in pediatric focal epilepsy: impact of underlying etiology. *Epilepsy Behav.* 2014;**34**:20-4.

11. Suleiman J, Wright S, Gill D, Brilot F, Waters P, Peacock K, et al. Autoantibodies to neuronal antigens in children with new-onset seizures classified according to the revised ILAE organization of seizures and epilepsies. *Epilepsia.* 2013;**54**(12):2091-100.

12. Wirrell E, Wong-Kisiel L, Mandrekar J, Nickels K. Predictors and course of medically intractable epilepsy in young children presenting before 36 months of age: a retrospective, population-based study. *Epilepsia.* 2012;**53**(9):1563-9.

13. Sierra-Marcos A, Toledo M, Quintana M, Edo MC, Centeno M, Santamarina E, et al. Diagnosis of epileptic syndrome after a new onset seizure and its correlation at long-term follow-up: longitudinal study of 131 patients from the emergency room. *Epilepsy Res.* 2011;**97**(1-2):30-6.

14. Wirrell EC, Grossardt BR, Wong-Kisiel LC, Nickels KC. Incidence and classification of new-onset epilepsy and epilepsy syndromes in children in Olmsted County, Minnesota from 1980 to 2004: a population-based study. *Epilepsy Res.* 2011;**95**(1-2):110-8.

15. Hsieh D, Chang T, Tsuchida T, Vezina L, Vanderver A, Siedel J, et al. New-onset afebrile seizures in infants: role of neuroimaging. *Neurology.* 2010;**74**(2):150-6.

16. Chen C-Y, Chang Y-J, Wu H-P. New-onset seizures in pediatric emergency. *Pediatrics & Neonatology.* 2010;**51**(2):103-11.

17. Geerts A, Arts WF, Stroink H, Peeters E, Brouwer O, Peters B, et al. Course and outcome of childhood epilepsy: a 15-year follow-up of the Dutch Study of Epilepsy in Childhood. *Epilepsia.* 2010;**51**(7):1189-97.

18. Jones JE, Watson R, Sheth R, Caplan R, Koehn M, Seidenberg M, et al. Psychiatric comorbidity in children with new onset epilepsy. *Dev Med Child Neurol.* 2007;**49**(7):493-7.

19. Doescher JS, deGrauw TJ, Musick BS, Dunn DW, Kalnin AJ, Egelhoff JC, et al. Magnetic resonance imaging (MRI) and electroencephalographic (EEG) findings in a cohort of normal children with newly diagnosed seizures. *Journal of child neurology.* 2006;**21**(6):490-5.

20. Wheless JW, Neto W, Wang S, Group E-S. Topiramate, carbamazepine, and valproate monotherapy: double-blind comparison in children with newly diagnosed epilepsy. *J Child Neurol.* 2004;**19**(2):135-41.

21. Sztriha L, Gururaj AK, Bener A, Nork M. Temporal lobe epilepsy in children: Etiology in a cohort with new‐onset seizures. *Epilepsia.* 2002;**43**(1):75-80.

22. Shinnar S, Berg AT, Moshe SL, Shinnar R. How long do new‐onset seizures in children last? *Annals of neurology.* 2001;**49**(5):659-64.
